# Effects of bromelain on endocannabinoid system and oxidative stress in a rat model of endometriosis

**DOI:** 10.1590/1806-9282.20260396

**Published:** 2026-09-21

**Authors:** Irem Herguner, Taylan Onat, Emin Kaymak, Zeliha Nur Ergül, Seyma Fadiloglu

**Affiliations:** 1 Sincan Training and Research Hospital, Clinic of Obstetrics and Gynecology – Ankara, Türkiye.; 2 Yozgat Bozok University, Faculty of Medicine, Department of Histology and Embryology – Yozgat, Türkiye.

**Keywords:** Endometriosis, Endocannabinoids, Oxidative stress, Bromelain

## Abstract

**OBJECTIVE:**

The objective of this study is to evaluate the effects of bromelain on serum endocannabinoid levels and oxidative stress markers in a rat model of surgically induced endometriosis.

**METHODS:**

A total of 24 female Wistar albino rats were randomly assigned to three groups (n=8 each): Sham, endometriosis, and endometriosis treated with bromelain (endometriosis+B). Endometriosis was induced by autotransplantation of uterine tissue. Bromelain (20 mg/kg/day) was administered orally for 15 consecutive days. Serum levels of 2-arachidonoylglycerol, anandamide, superoxide dismutase, glutathione, and malondialdehyde were measured using ELISA. Statistical analysis was performed using one-way analysis of variance followed by Tukey's post hoc test.

**RESULTS:**

Induction of endometriosis significantly reduced serum 2-arachidonoylglycerol, anandamide, superoxide dismutase, and glutathione levels while increasing malondialdehyde concentrations compared with the Sham group (p<0.05). Bromelain treatment significantly improved most endocannabinoid and oxidative stress parameters compared with the untreated endometriosis group (p<0.05).

**CONCLUSION:**

Bromelain ameliorated endocannabinoid deficiency and oxidative stress in experimental endometriosis. These findings suggest that bromelain may represent a promising non-hormonal therapeutic candidate targeting interconnected inflammatory and redox pathways in endometriosis.

## INTRODUCTION

Endometriosis is a chronic, estrogen-dependent inflammatory disorder characterized by the presence of endometrial-like tissue outside the uterine cavity, affecting approximately 10% of reproductive-aged women^1^. It is associated with chronic pelvic pain, dysmenorrhea, dyspareunia, and infertility, significantly impairing quality of life. Despite its high prevalence, the pathophysiology of endometriosis remains incompletely understood, and current treatments are limited by recurrence and adverse effects.

The establishment and persistence of endometriotic lesions involve complex interactions between hormonal imbalance, immune dysregulation, angiogenesis, and oxidative stress. Increased production of pro-inflammatory cytokines and reactive oxygen species contributes to tissue proliferation and resistance to apoptosis^2^. More recently, the endocannabinoid system (ECS) has emerged as a potential regulator of inflammation, pain modulation, and reproductive function. Endocannabinoids such as anandamide and 2-arachidonoylglycerol (2-AG) are involved in immune regulation and nociceptive signaling, and dysregulation of this system has been implicated in gynecological disorders, including endometriosis^3^. However, the relationship between oxidative stress and endocannabinoid signaling in endometriosis remains insufficiently explored.

Given the multifactorial nature of endometriosis and the limitations of current therapies, there is growing interest in investigating novel agents with anti-inflammatory and antioxidant properties. Bromelain, a proteolytic enzyme complex derived from Ananas comosus, has demonstrated anti-inflammatory, anti-edematous, and antioxidant effects in experimental and clinical studies^4–6^. Nevertheless, its potential effects on endocannabinoid modulation and oxidative stress in endometriosis have not yet been fully elucidated.

Therefore, this study aimed to evaluate the effects of bromelain on serum endocannabinoid levels and oxidative stress markers in an experimental rat model of endometriosis.

## METHODS

### Animals and experimental design

A total of 24 female Wistar albino rats (8–10 weeks old, weighing 180–220 g) were obtained from the Experimental Animal Production Laboratory. The animals were housed in standard plastic cages under controlled conditions (21±4°C, 60±5% relative humidity, 12-h light/dark cycle) with free access to commercial rat feed and water ad libitum. All experimental procedures were approved by the Local Ethics Committee for Animal Experiments (Decision No: 18, dated 02/10/2020).

Rats were randomly divided into three groups (n=8 per group):

Group 1 (Sham): Control group, no surgical intervention
Group 2 (EMS): Endometriosis-induced group
Group 3 (EMS+B): Endometriosis-induced+Bromelain-treated group (20 mg/kg/day, oral gavage for 15 days).

### Induction of endometriosis

Endometriosis was surgically induced using a modified autotransplantation method. Briefly, rats were anesthetized with an intraperitoneal injection of ketamine (50 mg/kg) and xylazine (10 mg/kg). A midline laparotomy was performed, and two 4×4 mm uterine tissue fragments were excised from the donor uterine horns. Each tissue fragment was sutured to the right and left lateral abdominal walls using 6-0 Prolene sutures. The abdominal wall and skin were closed with 3-0 silk sutures. Postoperative analgesia was provided with carprofen (5 mg/kg, subcutaneously). Sham-operated animals underwent laparotomy without uterine tissue transplantation, followed by abdominal wall closure identical to that performed in the endometriosis groups.

### Bromelain treatment

Starting from day 15 after surgery, rats in the EMS+B group received bromelain (20 mg/kg/day) via oral gavage for 15 consecutive days. The selected dose was based on previously published experimental studies evaluating the biological effects and therapeutic potential of bromelain in rat models^7^. The treatment duration was chosen to encompass approximately three estrous cycles in rats and to allow evaluation of bromelain-induced biochemical changes. The sham and EMS groups received an equivalent volume of distilled water.

### Blood collection

On day 30, all rats were euthanized by cervical dislocation under deep anesthesia. Blood samples were collected via cardiac puncture and centrifuged at 3,000 rpm for 10 min to obtain serum, which was stored at −80°C for biochemical analysis.

### Biochemical analysis

Serum levels of 2-AG, anandamide, superoxide dismutase (SOD), glutathione (GSH), and malondialdehyde (MDA) were measured using commercially available ELISA kits according to the manufacturer's instructions.

The following ELISA kits were used:

- Rat 2-AG (Cat. No: E1322Ra, Bioassay Technology Laboratory, Shanghai, China)
- General Anandamide (Cat. No: EA0024Ge, Bioassay Technology Laboratory, Shanghai, China)
- Rat SOD (Cat. No: EA0168Ra, Bioassay Technology Laboratory, Shanghai, China)
- Rat GSH (Cat. No: EA0113Ra, Bioassay Technology Laboratory, Shanghai, China)
- Rat MDA (Cat. No: EA0156Ra, Bioassay Technology Laboratory, Shanghai, China)

Absorbance was read at 450 nm using an ELISA reader.

### Statistical analysis

All statistical analyses were carried out using the GraphPad Prism version 7.00 for Mac (GraphPad Software, La Jolla, CA). The D’Agostino-Pearson omnibus test was used to identify the normal distribution of the data. In the case of normal distribution, quantitative variables were compared using a one-way analysis of variance (ANOVA) followed by Tukey's post hoc test for multiple comparisons. The data were expressed as the mean of normalized data±standard deviation of the mean. p<0.05 was considered statistically significant.

## RESULTS

**Figure 1:**
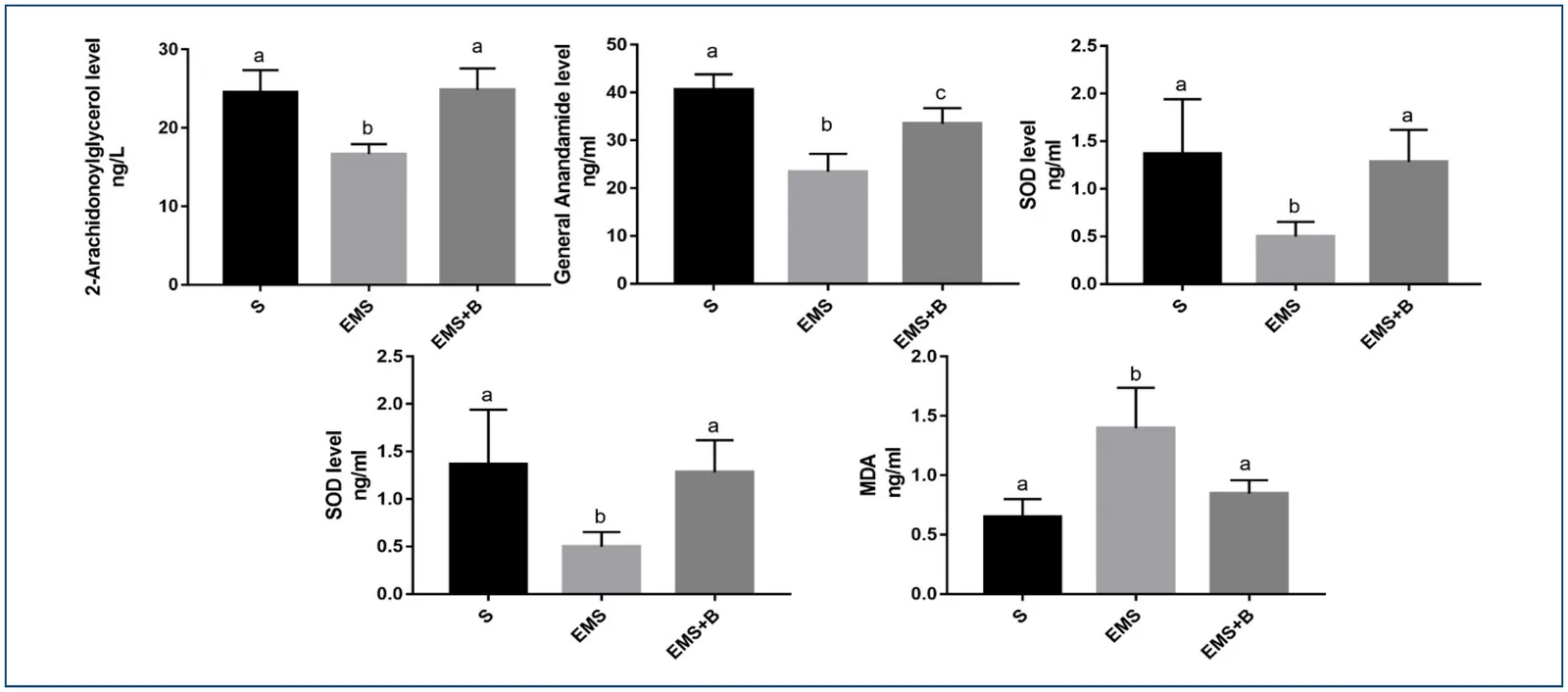
Effects of bromelain treatment on serum endocannabinoid and oxidative stress markers in experimental endometriosis. Groups sharing the same letter are not significantly different, whereas groups with different letters differ significantly according to Tukey's post-hoc test following one-way ANOVA (p<0.05).

Serum biochemical analysis demonstrated significant alterations in endocannabinoid and oxidative stress markers among the experimental groups (Table 1 and Figure 1).

**Table 1:** Serum endocannabinoid and oxidative stress markers in sham, endometriosis, and bromelain-treated endometriosis groups.

Parameter	Sham (n=8)	EMS (n=8)	EMS+B (n=8)	p-value	p-value Sham-EMS	p-value Sham-EMS+B	p-value EMS-EMS+B
2-AG (ng/mL)	0.025±0.0029	0.017±0.0013	0.025±0.0027	**<0.001**	**<0.001**	0.975	**<0.001**
Anandamide (ng/mL)	40.59±3.22	23.43±3.76	33.50±3.23	**<0.001**	**<0.001**	**0.009**	**0.001**
SOD (ng/mL)	1.36±0.57	0.49±0.15	1.28±0.33	**0.011**	**0.015**	0.942	**0.024**
GSH (mg/L)	316.60±64.94	214.80±34.86	275.30±19.20	**0.007**	**0.006**	0.317	0.091
MDA (ng/mL)	0.64±0.15	1.39±0.34	0.84±0.11	**<0.001**	**<0.001**	0.370	**0.002**

Values are presented as mean±standard deviation (SD). Overall group differences were assessed using one-way analysis of variance. Pairwise comparisons between groups (Sham vs. EMS, Sham vs. EMS+B, and EMS vs. EMS+B) were performed using Tukey's post-hoc test. 2-AG: 2-arachidonoylglycerol; EMS: endometriosis; SOD: superoxide dismutase; GSH: glutathione; MDA: malondialdehyde. Bold values indicate statistical significance (p<0.05).

### Endocannabinoid levels

Serum 2-AG levels were significantly reduced in the EMS group compared with the Sham group (16.67±1.25 vs. 24.52±2.85 ng/L, p<0.001). Bromelain treatment improved 2-AG concentrations in the EMS+B group (24.84±2.73 ng/L), which were significantly higher than those in the EMS group (p<0.001) and comparable to Sham levels (p>0.05).

Similarly, anandamide levels were markedly decreased in the EMS group compared with Sham (23.43±3.76 vs. 40.59±3.22 ng/mL, p<0.001). Administration of bromelain significantly increased anandamide levels in the EMS+B group (33.50±3.23 ng/mL, p<0.001 vs. EMS); however, levels remained significantly lower than in the Sham group (p<0.05).

### Oxidative stress markers

SOD levels were significantly decreased in the EMS group compared with Sham (0.49±0.15 vs. 1.36±0.57 ng/mL, p<0.01). Bromelain treatment significantly increased SOD levels relative to the EMS group (1.28±0.33 ng/mL, p<0.01), with no significant difference compared with Sham (p>0.05).

A similar pattern was observed for GSH. EMS significantly reduced GSH levels compared with Sham (214.80±34.86 vs. 316.60±64.94 mg/L, p<0.01). The EMS+B group showed significantly higher GSH levels than EMS (275.30±19.20 mg/L, p<0.05), without a statistically significant difference compared with Sham (p>0.05).

In contrast, MDA, a marker of lipid peroxidation, was significantly elevated in the EMS group compared with Sham (1.39±0.34 vs. 0.64±0.15 ng/mL, p<0.001). Bromelain administration significantly reduced MDA levels in the EMS+B group (0.84±0.11 ng/mL, p<0.001 vs. EMS), restoring values to levels comparable with the Sham group (p>0.05).

## DISCUSSION

Endometriosis is a chronic, estrogen-dependent inflammatory disease affecting approximately 10% of reproductive-aged women. Despite its high prevalence, the pathophysiology remains incompletely understood, and current treatment options are limited by side effects and recurrence rates. In this study, we established a surgically induced endometriosis model in rats and demonstrated that bromelain treatment significantly improved endocannabinoid levels and ameliorated oxidative stress markers, highlighting its potential as a multi-target therapeutic candidate in endometriosis.

The surgical autotransplantation model used in this study, first described by Vernon and Wilson in 1985, remains the most widely employed experimental approach for studying endometriosis pathophysiology and therapeutic interventions^8,9^. Our biochemical findings demonstrated significant reductions in serum 2-AG and anandamide (AEA) levels following endometriosis induction, consistent with clinical observations in women with endometriosis^10^. Andrieu et al. reported significantly lower peritoneal fluid concentrations of AEA and 2-AG in affected women and identified a negative correlation between endocannabinoid levels and pain intensity, suggesting that endocannabinoid deficiency may contribute to pain sensitization mechanisms in endometriosis^11^.

The distinct biological roles of AEA and 2-AG warrant consideration. AEA preferentially activates CB1 receptors involved in nociceptive pathways, whereas 2-AG acts as a full agonist at both CB1 and CB2 receptors and plays a role in immune modulation^11,12^. The concurrent reduction of both mediators observed in our model suggests a broader dysregulation of the ECS rather than selective impairment of a single signaling axis.

Emerging evidence implicates the ECS in angiogenesis and cellular survival. While CB1 activation may promote neovascularization, CB2 signaling appears to exert inhibitory effects on aberrant angiogenesis^13^. Reduced endocannabinoid tone may contribute to lesion persistence through impaired regulation of inflammatory and survival pathways.

Consistent with previous literature, our results demonstrated significant oxidative stress in the EMS group, characterized by decreased SOD and GSH levels and elevated MDA concentrations^14^. These findings reinforce the concept that redox imbalance is a central component of endometriosis pathophysiology and may interact with ECS dysregulation to perpetuate inflammation and lesion persistence.

Recent clinical discourse has questioned the routine surgical management of endometriomas. The Reproductive BioMedicine Online (RBMO) review by Urman et al.^15^ emphasizes that surgical intervention should be carefully individualized due to risks including diminished ovarian reserve, recurrence, and procedural complications. In parallel, hormonal therapies, although effective in symptom control, are associated with adverse effects such as bone mineral density loss, mood changes, and contraceptive consequences that may be undesirable in women seeking fertility^16^. Nonsteroidal anti-inflammatory drugs (NSAIDs) offer symptomatic relief but do not modify disease progression.

Beyond conventional medical therapies, several antioxidant and anti-inflammatory compounds have demonstrated beneficial effects in experimental endometriosis models. These studies provide a broader context for understanding the potential role of oxidative stress modulation as a therapeutic strategy in endometriosis. For example, resveratrol has been shown to inhibit vascularization and cell proliferation through modulation of VEGF and matrix metalloproteinases and to reduce oxidative stress by enhancing antioxidant enzyme activity while decreasing MDA levels^17–19^. Similarly, curcumin exhibits anti-inflammatory and anti-angiogenic properties in endometriosis models through NF-κB and COX-2 inhibition^20,21^. Omega-3 polyunsaturated fatty acids have also shown promise in reducing endometriosis-associated pain and inflammation through modulation of prostaglandin synthesis^22^. More recently, platelet-rich plasma (PRP) has emerged as a potential therapeutic approach, with experimental studies showing reduced lesion size and inflammation in rat models of endometriosis^23,24^.

Bromelain treatment significantly improved endocannabinoid levels and antioxidant capacity in our study. These beneficial effects can be attributed to bromelain's multifaceted pharmacological properties. Kansakar et al.^6^ described bromelain's ability to modulate NF-κB, MAPK, and COX-2 pathways, thereby reducing inflammatory cytokine production. Additionally, bromelain exerts antioxidant effects through free radical scavenging and upregulation of endogenous defense systems. Paksoy et al.^7^ demonstrated reduced oxidative stress indices in a rat ischemia-reperfusion model following bromelain administration.

Although previous studies have investigated bromelain in combination with other antioxidant agents such as N-acetyl cysteine (NAC) and alpha-lipoic acid (LA), data on its isolated use in endometriosis are lacking. In an experimental study, the combined administration of NAC, LA, and bromelain demonstrated significant anti-inflammatory and proapoptotic effects, including reduced cyst number and size as well as suppression of endothelial activation markers such as VCAM-1^25^. Similarly, clinical evidence from the LEAP study showed that an antioxidant preparation containing NAC, LA, and bromelain significantly improved endometriosis-associated pelvic pain and reduced the need for analgesics^26^. These findings suggest a potential synergistic interaction between antioxidant compounds in modulating inflammation and oxidative stress pathways.

However, unlike these combination therapies, the present study specifically evaluates bromelain as a single agent. Because these studies evaluated bromelain as part of a combination therapy, their findings cannot be directly extrapolated to bromelain monotherapy. To the best of our knowledge, this is the first study demonstrating that isolated bromelain administration can improve both endocannabinoid balance and oxidative stress parameters in an experimental endometriosis model. These findings provide preclinical evidence supporting bromelain as a potential non-hormonal therapeutic strategy targeting multiple interconnected mechanisms, including ECS dysfunction, oxidative stress, and inflammation, without the adverse effects associated with hormonal therapies or surgical interventions.

However, several limitations must be acknowledged. First, although the rat model is widely used, it does not fully recapitulate human menstrual physiology or immune complexity. Second, formal blinding was not implemented during group allocation, treatment administration, or outcome assessment, which may have introduced a risk of observer-related bias. Third, the sham procedure consisted of laparotomy without uterine tissue transplantation and therefore may not have fully controlled for all effects related to surgical manipulation and inflammation. Fourth, histological confirmation of endometriotic lesions and quantitative assessment of lesion burden were not available for the present analysis, limiting the morphological characterization of the experimental model and the ability to correlate biochemical findings with disease severity. We also did not evaluate behavioral pain parameters, which would enhance translational interpretation. In addition, the relatively small sample size may have limited statistical power to detect modest effect sizes and increased the risk of type II error. Furthermore, optimal dosing strategies and long-term safety profiles remain to be defined. Esterase or enzyme inhibitors were not used during blood collection and processing; therefore, ex vivo degradation of endocannabinoids cannot be completely excluded. Furthermore, endocannabinoid levels were measured using ELISA rather than liquid chromatography–tandem mass spectrometry (LC-MS/MS), which is considered the gold-standard analytical method for quantification.

Finally, the precise molecular mechanisms underlying bromelain's effects on ECS enzymes (fatty acid amide hydrolase [FAAH] and monoacylglycerol lipase [MAGL]), receptor expression, and related signaling pathways warrant further investigation. Future studies should incorporate behavioral assessments, histological quantification, bromelain-only control groups, and mechanistic analyses to better characterize the isolated effects of bromelain and clarify its therapeutic positioning. Comparative and combination studies with established medical treatments would further delineate its role within the evolving endometriosis management paradigm.

## CONCLUSION

Surgically induced endometriosis in rats was associated with significant endocannabinoid deficiency and oxidative stress, both of which were ameliorated by bromelain treatment. These findings support the hypothesis that ECS dysregulation and redox imbalance represent interrelated components of endometriosis pathogenesis. The ability of bromelain to improve endocannabinoid tone and antioxidant capacity supports further investigation of bromelain as a potential non-hormonal therapeutic approach in endometriosis.

If future clinical studies confirm these findings, bromelain could eventually be considered as a potential adjunctive strategy, particularly in patients seeking non-hormonal or fertility-preserving options. Well-designed clinical trials are required to determine its efficacy in reducing pain, improving quality of life, and potentially influencing reproductive outcomes in women with endometriosis.

## Data Availability

The datasets generated and/or analyzed during the current study are available from the corresponding author upon reasonable request.
